# Supplementary material for: Enhanced Light Absorption and Efficient Carrier Collection in MoS2 Monolayers on Au Nanopillars
Source: Nanomaterials (Basel). 2022 May 5;12(9):1567. doi: 10.3390/nano12091567 (PMC9104364; doi:10.3390/nano12091567)
Supplement: Supplementary file 1 [file nanomaterials-12-01567-s001.zip › nanomaterials-1715199-supplementary.pdf]

nanomaterials

Supplementary Materials

Enhanced Light Absorption and Efficient Carrier Collection in MoS_2 Monolayers on Au Nanopillars

Jungeun Song [1], Soyeong Kwon [1], Hyunjeong Jeong [1], Hyeji Choi [1], Anh Thi Nguyen [1], Ha Kyung Park [1], Hyeong-Ho Park [2], William Jo [1], Sang Wook Lee [1] and Dong-Wook Kim [1,*]

[1] Department of Physics, Ewha Womans University, Seoul 03760, Korea; sje10056996@gmail.com (J.S.); kwonsso91@gmail.com (S.K.); hjjeongssi@gmail.com (H.J.); hjchoi1214@gmail.com (H.C.); nthianh111@gmail.com (A.T.N.); hakyungpark@ewhain.net (H.K.P.); wmjo@ewha.ac.kr (W.J.); leesw@ewha.ac.kr (S.W.L.)

[2] Nanodevice Laboratory, Korea Advanced Nano Fab Center, Suwon 16229, Korea; hyeongho.park@kanc.re.kr

* Correspondence: dwkim@ewha.ac.kr

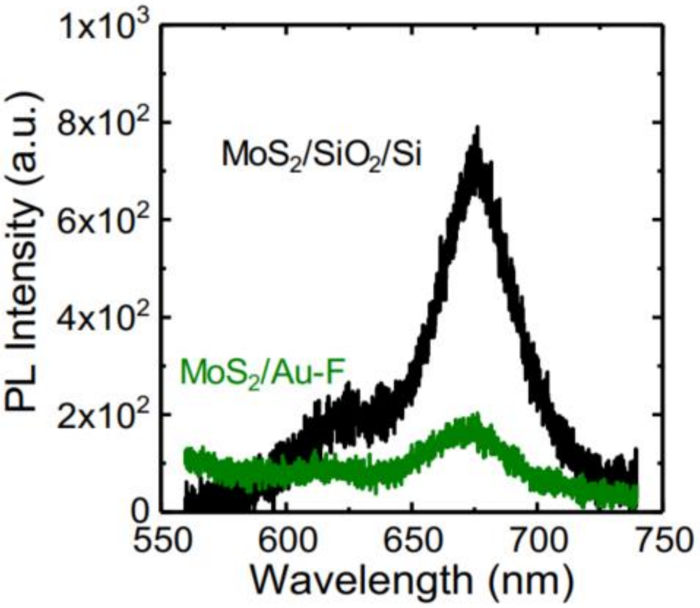

Figure S1. Typical PL spectra of pristine MoS_2 monolayers on SiO_2/Si wafers (MoS_2/SiO_2/Si, black) and MoS_2 monolayers transferred on Au thin films (MoS_2/Au-F, green). Au thin films significantly suppress the PL emission from MoS_2 monolayers on them.

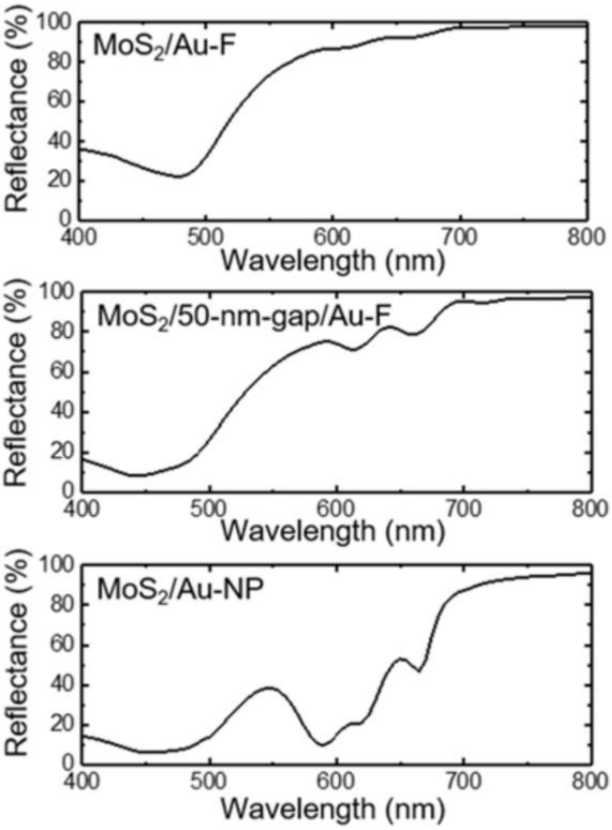

Figure S2. FDTD-calculated reflectance spectra of MoS_2 monolayers on Au(100 nm)/SiO_2/Si wafers without (MoS_2/Au-F) and with a 50-nm gap (MoS_2/50-nm-gap/Au-F). The latter shows reduced reflectance in broad wavelength range and clear dips at the exciton resonance wavelengths (615 and 660 nm). This suggests that the gap can enhance the optical absorption of the MoS_2 monolayers. The reflectance spectrum of MoS_2 monolayers on Au-NP is also compared with those of other two samples.

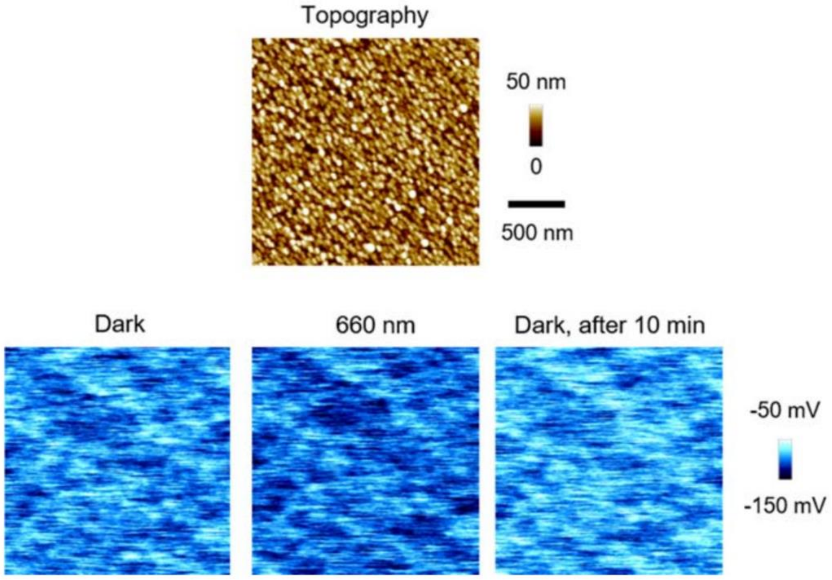

Figure S3. Surface topography and CPD maps of MoS_2/Au-F. The first dark-state CPD (CPD_D) map was obtained after storing the sample in dark for at least 1 hour. The CPD under light illumination (CPD_L) map was measured while shining 660-nm-wavelength light with the power density of 1.6

mW/cm² on the sample surface. The measured CPD_L is smaller than the measured CPD_D by several mV. The last CPD map in dark, obtained after leaving the sample in dark for 10 minutes, shows that the measured CPD returns to the initial dark state CPD.

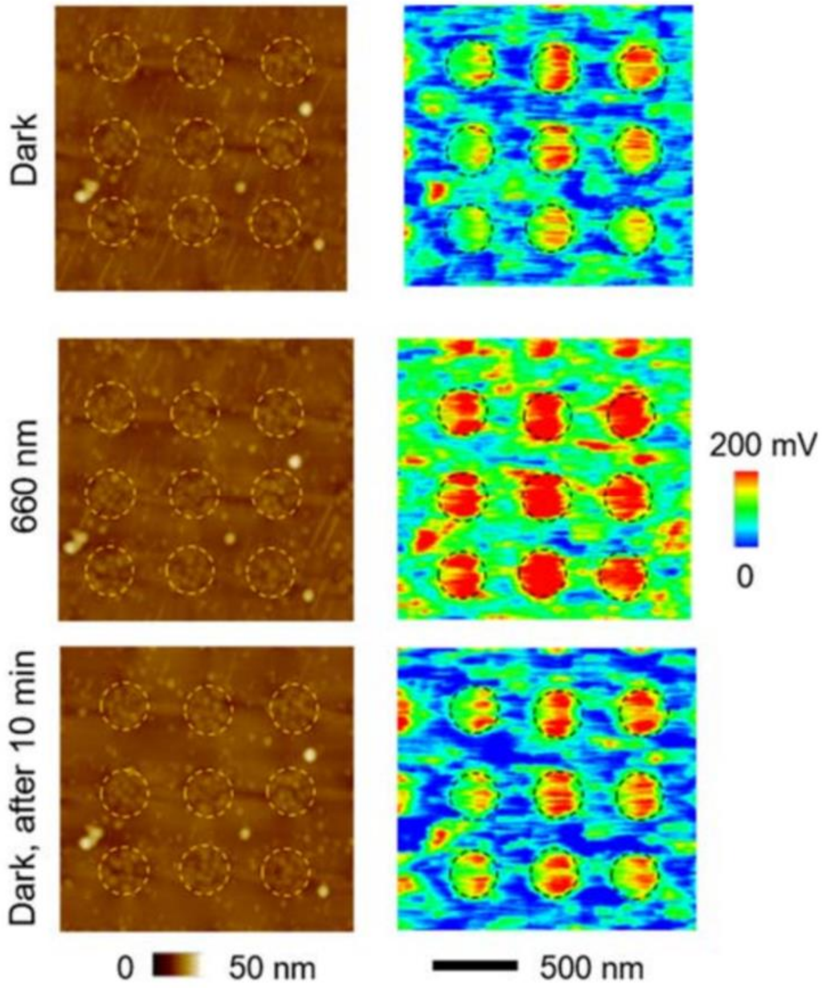

Figure S4. Surface topography (**left**) and CPD (**right**) maps of MoS_2/Au-NP. The maps at the top were taken in the initial dark state. The middle maps were obtained under illumination of 660-nm light (power density: 1.6 mW/cm²). The maps at the bottom were obtained after leaving the sample in dark for 10 minutes. The dashed line circles in the maps indicate the regions where the MoS_2 monolayers are in contact with the top surface of Au-NPs.
